# Molecular Dynamics Studies of Therapeutic Liquid Mixtures and Their Binding to Mycobacteria

**DOI:** 10.3389/fphar.2021.626735

**Published:** 2021-04-20

**Authors:** Hugo Monteiro, Filipa Santos, Alexandre Paiva, Ana Rita C. Duarte, Ricardo J. Ferreira

**Affiliations:** ^1^ LAQV, REQUIMTE, Chemistry Department of NOVA School of Science and Technology, Caparica, Portugal; ^2^ Red Glead Discovery AB, Lund, Sweden

**Keywords:** molecular dynamics, therapeutic liquid mixtures, mycobacteria, ethambuthol, tuberculosis

## Abstract

Tuberculosis is an highly contagious disease still considered by the WHO as one of most infectious diseases worldwide. The therapeutic approach, used to prevent and treat tuberculosis targets the *Mycobacterium tuberculosis* complex, comprises a combination of drugs administrated for long periods of time, which, in many cases, could cause several adverse effects and, consequently, low compliance of the patient to the treatment and drug-resistance. Therefore, therapeutic liquid mixtures formulated with anti-tuberculosis drugs and/or adjuvants in tuberculosis therapy are an interesting approach to prevent toxic effects and resistance to anti-tuberculosis drugs. The herein formulated therapeutic liquid mixtures, including ethambutol, arginine, citric acid and water under different molar ratios, were studied through a molecular dynamics approach to understand how ethambutol and arginine could be stabilized by the presence of citric acid and/or water in the mixture. To gain insights on how the uptake of these mixtures into the mycobacteria cell may occur and how a mycobacterial ABC transporter could contribute to this transport, multiple simultaneous ligand docking was performed. Interactions between citric acid and ethambutol involving the carboxyl and hydroxyl groups of citric acid with the amines of ethambutol were identified as the most critical ones. Water molecules present in the mixture provides the necessary network of hydrogen bonds that stabilize the mixture. Molecular docking additionally provided an interesting hypothesis on how the different mixture components may favor binding of ethambutol to an ABC importer. The data presented in this work helps to better understand these mixtures as well as to provide cues on the mechanisms that allow them to cross the mycobacterial cell membrane.

## Introduction

Tuberculosis persists as one of the major causes of morbidity and mortality caused by an infection, worldwide. It is an infectious disease triggered by *Mycobacterium tuberculosis* complex that is pathogenic in humans and animals and belongs to a group of bacteria named *Mycobacteria*. This group of bacteria are frequently resistant to several antibiotics, mostly due to slow absorption of the drugs through the highly hydrophobic mycobacterial cell envelope (Viveiros et al., 2003). The mycobacterial cell wall is composed by peptidoglycans, arabinogalactans, and mycolic acids (Torfs et al., 2019), which leads to the formation of an intrinsic barrier that hampers drugs penetration and can contribute to drug resistance to anti-tuberculosis drugs (Silva et al., 2013). In spite of a descending tendency observed on the evolution of tuberculosis over the last years, in 2019 the WHO reported 1.4 million deaths by this infectious disease (Fitzgerald et al., 2015; WHO, 2020). The treatment for tuberculosis includes a combination therapy of multiple antibiotics administrated for several months. One of the major problems in tuberculosis treatment is the emergence of multidrug resistance (MDR) that can occur during the therapy due to chromosomal mutations mechanisms (e.g., single nucleotide polymorphisms), drug-drug interactions, malabsorption of drugs and prolonged administration of multiple drugs, which in turn leads to a low compliance of the patient to the treatment (Forbes et al., 2018; Hameed et al., 2018).

Since this disease continues to present some gaps in the effective treatment, particularly when MDR occurs, new strategies are most welcome. In addition, optimization of the existing ones to reduce toxic and MDR associated events related to tuberculosis therapy may also play an important role in the treatment of the disease (WHO, 2015; Rendon et al., 2017; Sotgiu et al., 2017). One possible strategy to overcome these gaps is the development of shorter regimens of drug administration which could be achieved using alternative and sustainable solvents as therapeutic liquid mixtures (Aroso et al., 2016; Duarte et al., 2017). These mixtures are considered low transition temperature mixtures (LTTMs) and are formed mixing two or more components that at certain molar ratios, often form a clear viscous liquid at room temperature with a lower melting point than those of the initial compounds (Dai et al., 2013; Francisco et al., 2013; Aroso et al., 2016; Abbott et al., 2017). Different theories have been reported to explain which interactions could be involved to promote their stability as liquid forms and their properties. Accordingly, Abbott *et al.* had proposed hydrogen bonds as important contributors for the decrease of entropic differences of the phase transitions of the components and, concomitantly, for the reduction of their melting point (Abbott et al., 2017). The different atoms present in the molecules and the number of HBDs and HBAs are, hence, among the main factors that could determine the capacity of the different components of the mixture to interact, together with van der Waals and/or electrostatic forces (Abbott et al., 2017; Santana et al., 2019). In this sense, the charge delocalization could modulate the physicochemical properties of these eutectic liquid mixtures, when compared with initial components (Francisco et al., 2013). Furthermore, being water part of the system and acting as a component of the mixture, its presence usually facilitates the preparation and manipulation of the mixture by decreasing their viscosity and, quite often, increasing the stability of the mixture. However, adding more than 50% of water to the system could lead to a disruption of the hydrogen bonds and dilution of the components, creating a solution (Dai et al., 2015; Craveiro et al., 2016). In previous studies, the possibility of pH itself affect the solubility of other components in the mixture was explored and it was observed that the slight variation of pH did not affect the solubility of these eutectic mixtures and due to that, the pH was not considered in molecular dynamics (MD) studies (Santos et al., 2018; Roda et al., 2021).

In the pharmaceutical field, these mixtures have been used over the decades and several studies reported that the combination of active pharmaceutical ingredients (API) with compounds that could interact by van der Walls and electrostatic forces and/or hydrogen bonds, in determined molar ratios, could provide an enhancement of the some properties of the API such as solubility, permeability, and absorption (Evers et al., 1985; Stott et al., 1998; Aroso et al., 2015). One of the examples was reported by Stott and coworkers, they prepared eutectic mixtures combining ibuprofen with different terpenes and formed a therapeutic liquid mixture for enhancement of skin permeation (Stott et al., 1998). The therapeutic liquid mixtures comprise thousands of different combinations, and although their preparation is still based on an approach of trial and error, gaining insights about the interaction between the components of the mixture provides more information towards the successful development of these mixtures. The knowledge about how therapeutic liquid mixtures work and how they could be applied in pharmaceutical and biomedical field remains limited. However, it is a field that has been continuously explored, in an attempt to improve the characteristics of active pharmaceutical ingredients and enhance their pharmacokinetics and pharmacodynamics. To overcome these questions, MD represents an important tool that could boost the understanding of these mixtures, particularly how the components interact with each other to form a stable mixture (Kumari et al., 2018; Gutiérrez et al., 2019), by providing insights on the behavior of atoms and molecules and allowing the prediction of the interactions of a particular system during a pre-determined period of time. In the case of eutectic systems, this type of analysis could give insights about the chemical and physical properties of the systems, such as dynamics of the molecules, radial distribution functions (which describes how density varies as a function of distance from a reference particle), spatial distribution functions of the atoms, self-diffusion coefficients, intermolecular interaction, polar solvation energies, percentage of volume expansion and type of hydrogen-bonding interactions. To this matter, MD simulations had already proven its value by providing valuable knowledge regarding the solubility of active pharmaceutical ingredients (Gutiérrez et al., 2018, 2019) or through the study of the internal structure of deep eutectic solvents at its molecular level (Fetisov et al., 2018; Kumari et al., 2018).

In the current study, a MD approach was used to understand how an anti-tuberculosis drug, ethambutol (EMB), and a natural compound as arginine (ARG), described to act as an adjuvant in tuberculosis therapy (Schön et al., 2011; Farazi et al., 2015; Santos et al., 2018), could be stabilized by the presence of citric acid (CIT) and/or water in the mixture. Furthermore, a molecular docking study was also performed to unveil a possible CIT-promoted entry pathway for EMB through a recently published mycobacterial ABC transporter (Rempel et al., 2020).

## Materials and Methods

**FIGURE 1:**
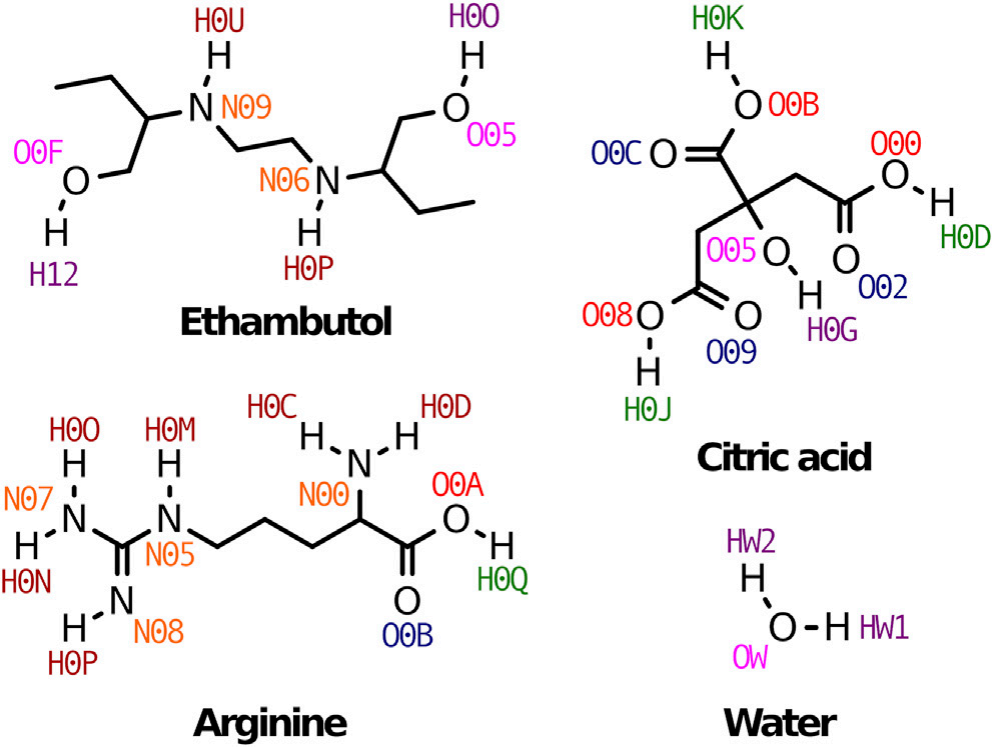
Therapeutic liquid mixtures components studied in this work. Atom names used through the manuscript are also indicated for clarity.

All molecules were parameterized in OPLS/AA force field (Jorgensen and Tirado-Rives, 1988) using LigParGen server (Jorgensen and Tirado-Rives, 2005; Dodda et al., 2017a; Dodda et al., 2017b) and are depicted in Figure 1. Four systems were considered for evaluation: citric acid monohydrate:arginine:water 1:1:7 (S1), citric acid monohydrate:ethambutol:water, 2:1:10 (S2) and 1:1:5 (S3), and citric acid monohydrate:ethambutol:arginine:water 2:1:1:7 (S4) (Table 1).

**TABLE 1:** Therapeutic liquid mixtures used for the molecular dynamics studies.

Therapeutic liquid mixture	Molar ratio	Molar fraction
Citric acid monohydrated:l-Arginine:H_2_O	1:1:7	0.11:0.11:0.78
Citric acid monohydrated:Ethambutol:H_2_O	2:1:10	0.15:0.077:0.77
Citric acid monohydrated:Ethambutol:H_2_O	1:1:5	0.14:0.14:0.71
Citric acid monohydrated:Ethambutol:l-Arginine:H_2_O	2:1:1:7	0.18:0.09:0.09:0.64

To avoid any bias due to system assembly, two smaller systems (S1 and S2) were initially built by creating a solvated box, replacing the number of water molecules by the species of interest and further removing the water molecules in excess to obtain the desired ratio. Both systems were energy minimized, with the system’s temperature being equilibrated using an NVT ensemble during 1-ns followed by a 1000-ns (1-µs) long MD simulation in an NpT ensemble. After this pre-equilibration procedure, larger boxes were generated by duplicating the initial box size in all xyz axis. The other systems (S3 and S4) were created by removing CIT (S3) or by adding ARG (S4), with the number of water molecules then adjusted to the desired ratio. Then, all systems were again simulated for 100 ps (NVT ensemble for temperature equilibration), followed by an 1-µs NpT ensemble run. Additional systems as ethambutol in water (EMB-H_2_O), citric acid in water (CIT-H_2_O) or arginine in water (ARG-H_2_O) were also built for comparison purposes and simulated in similar conditions as described above.

All MD simulations were performed using GROMACS 2020.3 (Pronk et al., 2013; Abraham et al., 2015). Molecule insertion and larger systems were obtained using internal GROMACS routines. While energy minimization steps used the steepest gradient method, all NVT ensemble runs for temperature equilibration used the velocity-rescale (V-rescale) thermostat (Bussi et al., 2007) and all NpT runs the Nosé-Hoover thermostat (310 K) and Parrinello-Rahman barostat (1 bar) (Nosé and Klein, 1983; Hoover, 1985; Nosé, 2002). In NVT ensemble runs, initial velocities were assigned from a Maxwell-Boltzmann distribution and periodic boundary conditions (PBC) were used in all simulations. To allow the calculation of non-bonded interactions in GPUs, a Verlet cut-off scheme was employed (Páll and Hess, 2013). All bond lengths were constrained using LINCS (Hess et al., 1997; Hess, 2008) (SETTLE for TIP5P waters) (Miyamoto and Kollman, 1992). All analysis were performed using the last 500-ns of the NpT ensemble MD simulation runs. Radial distribution functions (RDF) and spatial distribution functions (SDF) were calculated using GROMACS *gmx rdf* module and Volmap plugin in VMD (Humphrey et al., 1996), respectively. Images were rendered in VMD.

Multiple ligand simultaneous docking (MLSD) (Li and Li, 2010) was performed to evaluate the interaction of the different system’s components with a recently published mycobacterial ABC transporter, found to mediate hydrophilic compounds uptake (PDB ID: 6TQE) (Rempel et al., 2020). Standard molecular docking calculations were performed using a genetic algorithm (GA), in which the population size (ga_pop_size), the maximum number of energy evaluations (ga_num_eval) and the maximum number of generations (ga_num_generations) were set to 150, 5.000.000 and 25.000, respectively. The docking box, with a size of 48 × 68 × 62, grid spacing of 0.375 Å and centered at coordinates 119.15 × 118.874 × 112.419 (xyz coordinates) was defined to include a lower chamber separated from the cytosol by an arginine gate comprising a single Arg287 residues from both protomers. A total of 100 runs (ga_runs) were performed. Single docking using Autodock 4.2.6 was performed for comparison purposes, with ga_pop_size, ga_num_eval and ga_num_generation set to 150, 2.500.000 and 25.000, respectively. All other docking parameters were kept at default. Docking results clustering was performed using AutoDockTools (Morris et al., 2009) summarize_results4. py with a root-mean square tolerance of 10.

## Results and Discussion

### Molecular Dynamics Studies

Molecular dynamics is an unique tool that allows probing the internal structure of these therapeutic liquid mixtures and to gain insights on the interactions and configurations of each component at its molecular level (Kumari et al., 2018; Cao et al., 2019; Gutiérrez et al., 2019; Sapir and Harries, 2020). Herein, we employed MD simulations to study the molecular structure of each mixture, in order to gain insights on which chemical groups are involved in the formation of the eutectic mixture and to identify suitable chemical modifications that can further improve the properties of ethambutol, enabling them to be used as therapeutic delivery systems (Santos et al., 2018).

#### Hydrogen Bonding

**FIGURE 2:**
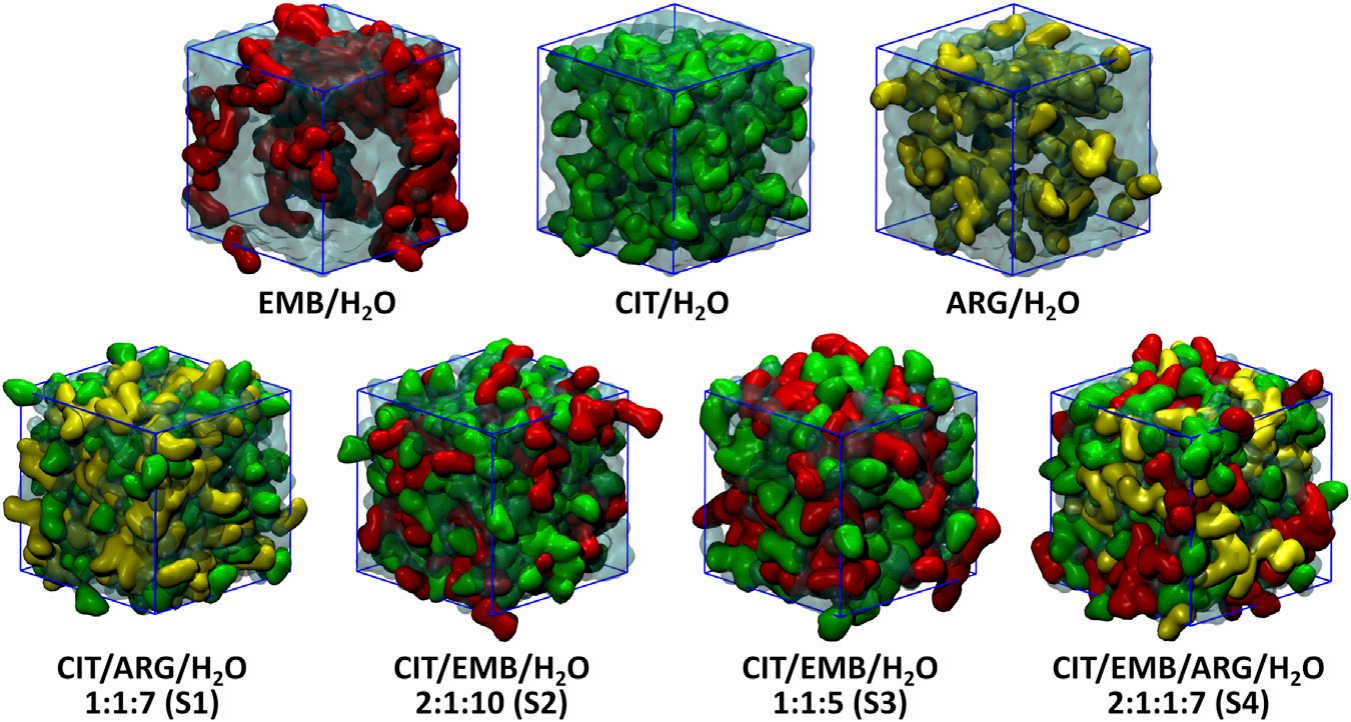
Final configuration (after 1 µs MD simulation time) for all studied systems.

**FIGURE 3:**
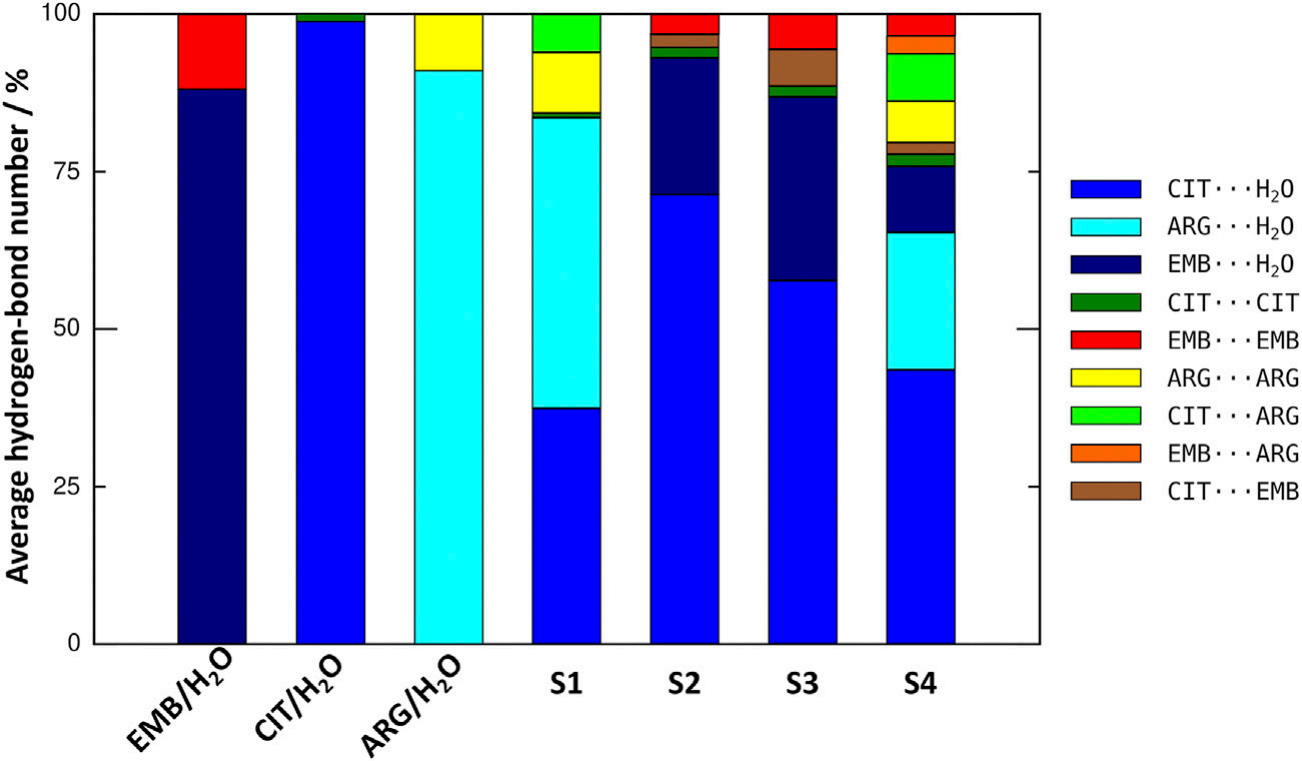
Comparison on the average number of hydrogen-bonds between all liquid mixtures components in the studied systems.

An initial set of MD simulations were performed comprising each of the liquid mixture component in water (Figure 2), namely EMB/H_2_O, CIT/H_2_O, and ARG/H_2_O. While no aggregation could be observed in either CIT/H_2_O or ARG/H_2_O systems, the system EMB/H_2_O remained inhomogeneous, with EMB molecules aggregated mostly due to intermolecular hydrogen-bonds (HBs) between the amino (pKa_1_ = 6.35 and pKa_2_ = 9.35) (Beggs and Andrews, 1974) and hydroxyl groups. Such intermolecular HBs account for nearly 12% of all calculated HBs, with the remaining 88% corresponding to the interactions between the second hydroxyl group and the surrounding water molecules (Figure 3). In the ARG/H_2_O systems a similar trend is observed (8% ARG-ARG vs. 92% ARG-H_2_O) however, in the CIT/H_2_O system almost all calculated HBs are between citric acid and water (99 vs. 1% CIT-CIT interactions).

When considering a liquid mixture as CIT/ARG/H_2_O (S1, Figure 3), most of all calculated HBs were observed between ARG/H_2_O and CIT/H_2_O pairs (46 and 37%, respectively). Quite interestingly, while intermolecular ARG-ARG hydrogen-bonding increases (10%), all novel ARG-CIT interactions between both components were accomplished by directly replacing water molecules of the first solvation shell by ARG. It seems that, even in a low percentage (6%), the newly established HBs are sufficient to improve ARG solubility and to drive S1 into a more homogeneous state when compared with ARG/H_2_O alone. For EMB-containing systems S2 and S3, again we observe that most of EMB/H_2_O HBs were replaced by the more favorable CIT/H_2_O interactions (71 and 51% of all calculated HBs for S2 and S3, respectively). In both systems, the number of HBs between EMB/CIT was low (2 and 6% for systems S2 and S3, respectively) but sufficient to improve the system’s homogeneity, as it can be seen by the absence of EMB aggregates in both S2 and S3 when compared with EMB/H_2_O mixture (Figure 3). Finally, in S4 the introduction of ARG replaces EMB/H_2_O interactions in a larger extent than of CIT/H_2_O, reaching similar values than those calculated for the S1 system regarding ARG/ARG, CIT/CIT, and ARG/CIT interactions (Figure 3). Therefore, the results seems to be in agreement with previous studies in which the increased solvation of CIT improves the solubility of both ARG and EMB (Santos et al., 2018). In the following section, we describe which chemical groups are responsible for the intermolecular interactions and which maintain their interactions with the surrounding environment.

#### Radial Distribution Functions of Therapeutic Liquid Mixtures Components

**FIGURE 4:**
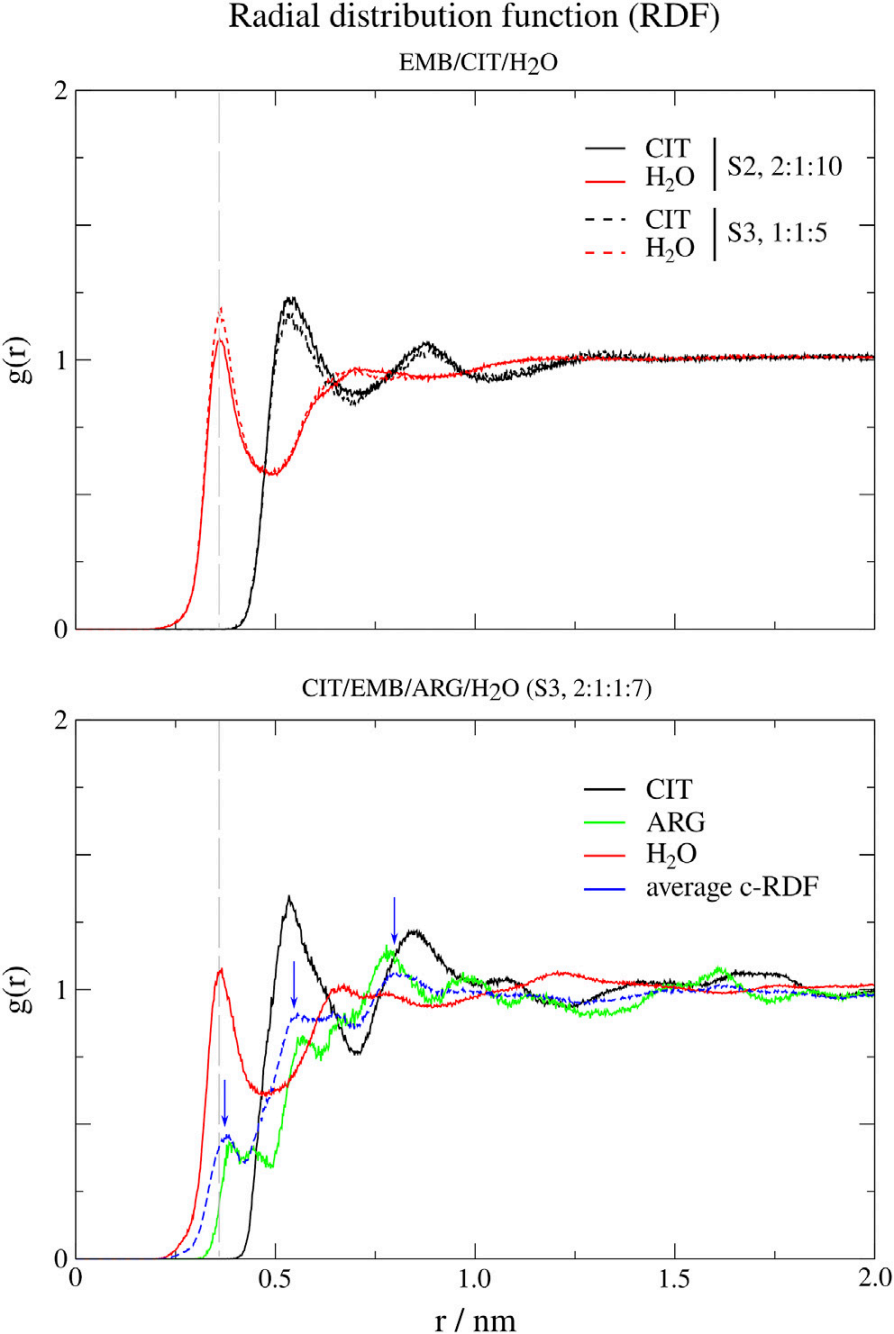
Center-of-mass radial distribution functions (c-RDFs) for citric acid (CIT), arginine (ARG), and water (H_2_O) around ethambutol (EMB) for systems S2-S4. Dashed grey line corresponds to the 0.35 nm cut-off for hydrogen bond calculations.

**FIGURE 5:**
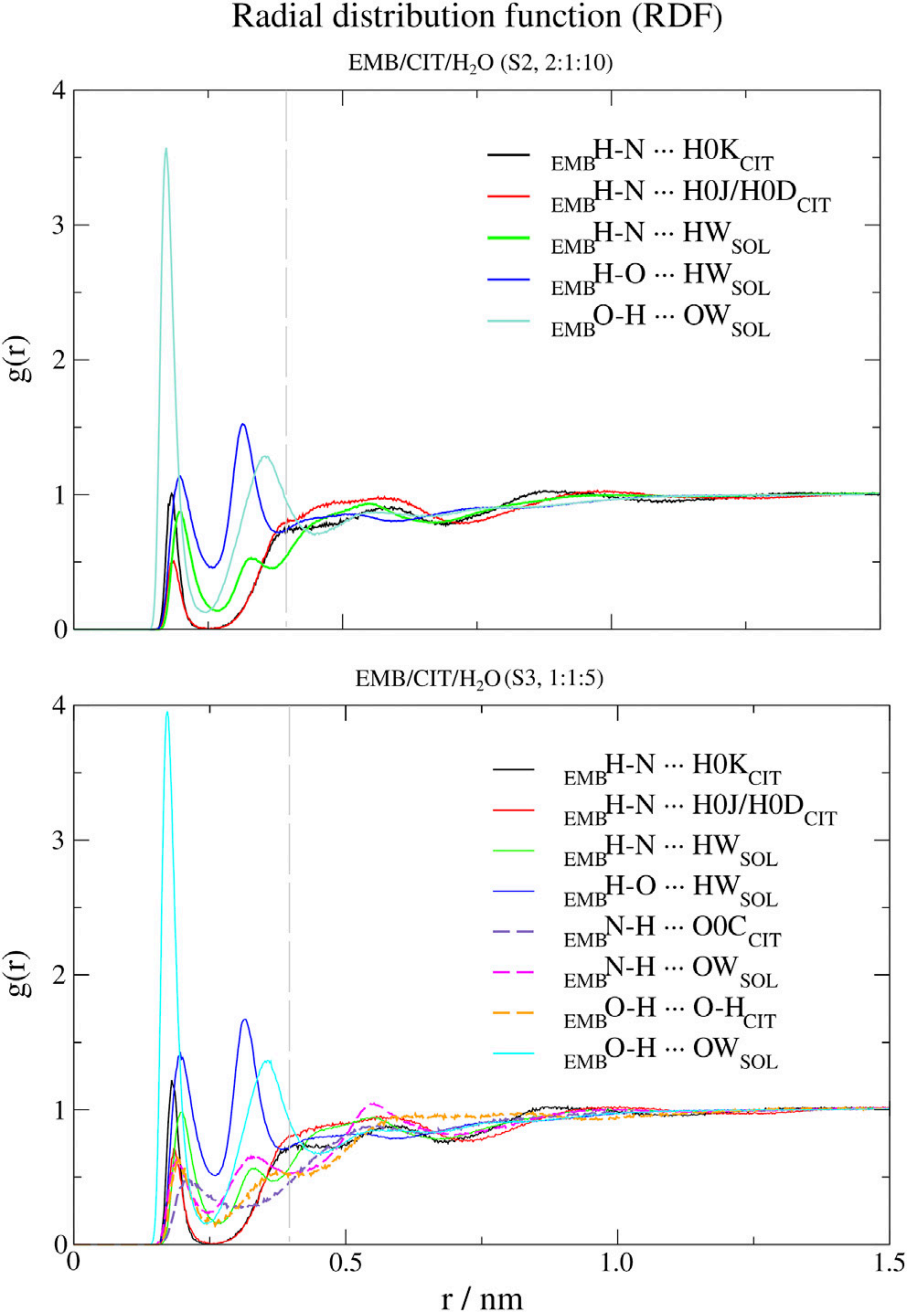
Atomic radial distribution functions (a-RDFs) between citric acid (CIT) and water (SOL) and ethambutol (EMB) for systems S2 and S3, in reference to ethambutol hydrogen-bond acceptor and donor groups. Dashed grey line corresponds to the 0.35 nm cut-off for hydrogen bond calculations.

**FIGURE 6:**
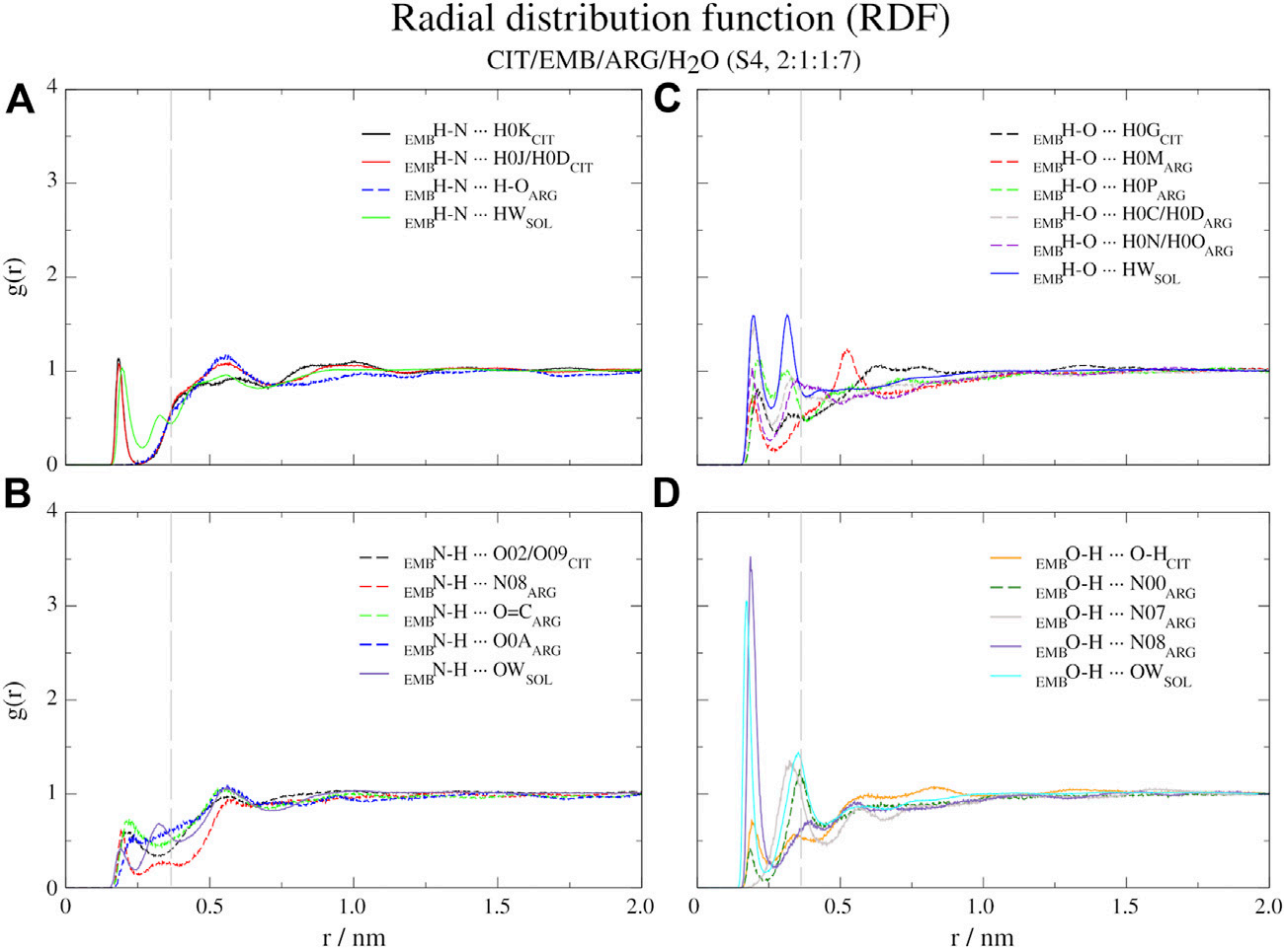
Atomic radial distribution functions for system S4, between ethambutol amine **(A)**, hydrogen-bond acceptor; **(B)**, hydrogen-bond donor and hydroxyl **(C)**, hydrogen-bond acceptor moieties; **(D)**, hydrogen-bond donor groups). Dashed grey line corresponds to the 0.35 nm cut-off for hydrogen bond calculations.

In order to probe the liquid mixtures structure of the different systems and observe the affinity between molecules that compose the mixtures, we calculated RDFs for both center-of mass (c-RDF, Figure 4) and atomic (a-RDF, Figures 5, 6) for all components of the mixture. Regarding c-RDF of systems S2 and S3, while changes in the components ratios only had minor alterations, the introduction of ARG (S4) was revealed by 1) the appearance of three peaks in the RDF profile at *r* = 0.38 nm (enabling intermolecular HB with EMB), *r* = 0.56 nm (HB with CIT) and *r* = 0.82 nm (also with CIT) and 2) by inducing a slight decrease in the water peak (when compared with S2) while reinforcing both citrates peaks at *r* = 0.54 nm and *r* = 0.85 nm. Therefore, the data suggests that by adding ARG, the structure of the liquid mixture is reinforced due to its ability to establish intermolecular interactions with both EMB and CIT molecules.

Next, we used a-RDF to better understand which specific interactions could be responsible for the interactions between the various liquid mixture components. From Figure 5 is possible to verify that, concerning direct interactions between CIT and EMB, the interactions were established between the hydrogen from carboxylic acid group at position C-2 (HBD) and EMB nitrogen atoms (HBA), meaning that R_2_H-N ··· H-O-CO_CIT_ in S2 system (CIT/EMB/H_2_O, 2:1:10; Figure 5) are two times more frequent than with any of the terminal carboxylic acid groups. Thus, the latter will remain available to mediate interactions with the surrounding aqueous environment. However, as titration studies show that EMB has two dissociation constants (Beggs and Andrews, 1974), we also expected that only one amine group undergoes hydrogen-bonding with CIT in the former. This does not change in S3 (CIT/EMB/H_2_O, 1:1:5), nonetheless the change in the liquid mixture components ratio promoted additional interactions, via hydrogen-bonding, between the carbonyl moiety of carboxylic acid at C-2 and the hydrogen atoms bound to the nitrogen atoms (R_2_N-H ··· O=C-OH), between the amine moiety and water molecules (R_2_N-H ··· OH_2_) and between the hydroxyl groups of CIT and EMB (R_2_N-H ··· OH-R). Regarding the hydroxyl groups of EMB, in both systems only interactions with water molecules were observed, either as hydrogen-bond donor or acceptor.

Regarding S4 (CIT/EMB/ARG/H_2_O, 2:1:1:7), the addition of ARG to form a new system fundamentally changed the interaction modes of EMB with the surrounding environment but not with CIT (Figure 6). While the interaction of CIT and EMB was maintained and even reinforced, as it can be seen from the sharper peaks at 0.19 nm in Figure 6A, additional interactions between the amine group of EMB (HBA) and the carboxylic acid (HBD) moiety of ARG (R_2_H-N ··· H-O-CO_ARG_) were observed. Other pairs, although less frequent than the former, could also be observed in which the EMB amine group acted as a HBD (Figure 6B). Concerning EMB hydroxyl groups, they act preferentially as HBDs (Figures 6C vs. 6D). Quite remarkably, ARG appears to be able to disrupt the HB network of water molecules observed in all former systems, as it can be seen by the appearance of a sharp and very intense peak slightly shifted from that of water (*r* = 0.17 vs. 0.19 nm), assigned to the hydrogen bonding between the hydroxyl and the guanidinium nitrogen N08, and two additional peaks at *r* = 0.32 nm (guanidinium nitrogen N07) and *r* = 0.36 nm (α-amino group of ARG).

The results obtained with MD simulations allowed us to understand that 1) only one carboxylic group of CIT is essential to promote interactions with the amine groups of EMB, and that 2) ARG, although also interacting with the same amine groups, prefers to establish intermolecular HBs with EMB hydroxyl groups. Therefore, it is conceivable that both CIT and ARG provide a greater balance between hydrophobicity and hydrophilicity when interacting with EMB, while the remaining functional groups continue immersed in the aqueous environment structuring the therapeutic liquid mixture.

#### Spatial Distribution Functions

**FIGURE 7:**
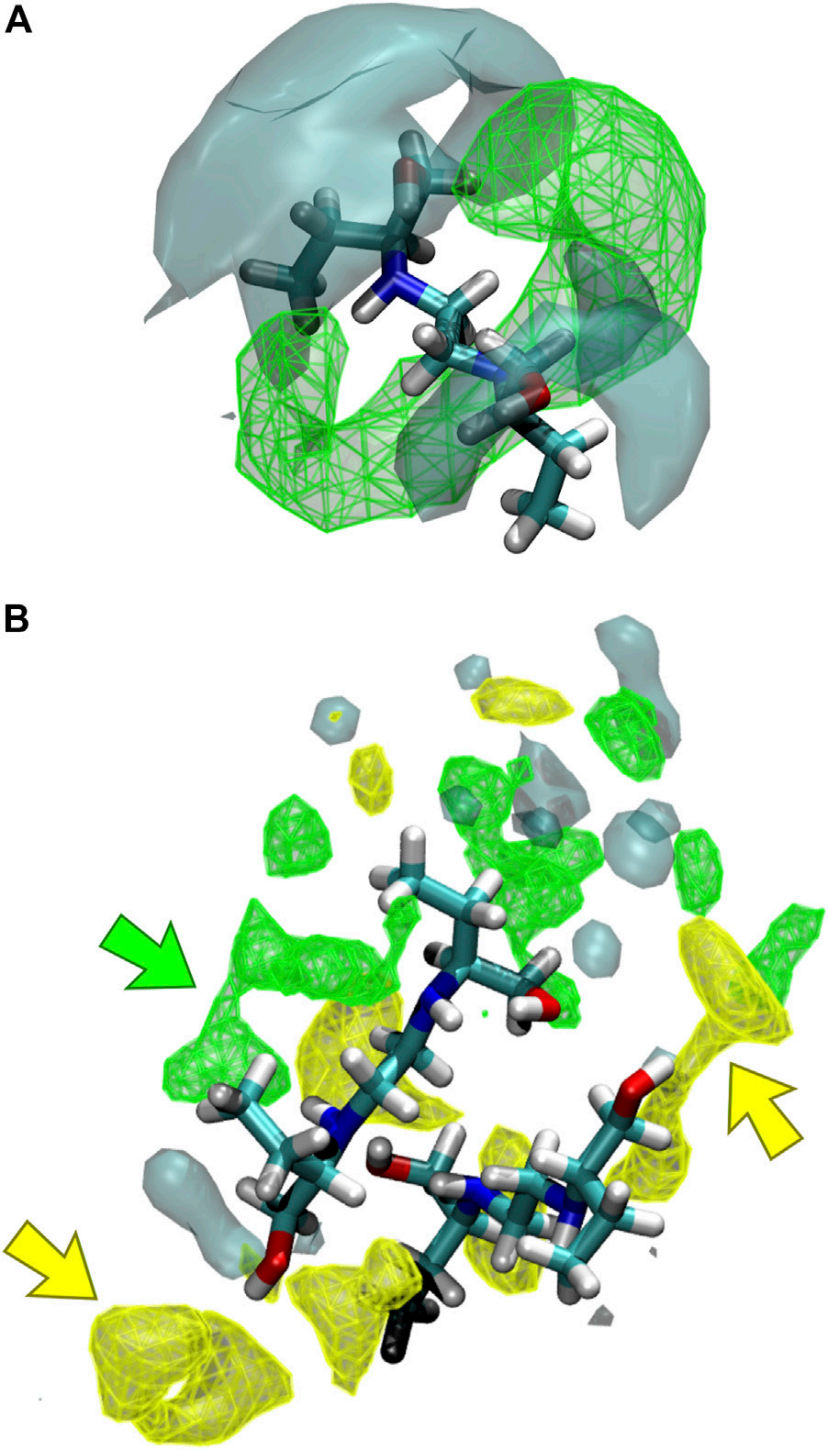
Spatial distribution functions (SDFs) of the liquid mixtures components around ethambutol for **(A)** S2 and S3, and **(B)** S4 systems. Specific interactions between citric acid-ethambutol and arginine-ethambutol are identified in the latter by green and yellow arrows, respectively.

The interaction of the different liquid mixture components can also be observed from the corresponding SDF. Herein, SDF was used to determine specific interactions between the molecules, using a three-dimensional density distribution of CIT, ARG, and water molecules in a local coordinate system linked with ethambutol. From Figure 7A, it is possible to see that CIT mainly occupies a toroidal section around the amine groups of EMB, which is in agreement with results depicted in the RDF plots. Hence, increased water densities are only found in the vicinity of the hydroxyl groups of EMB, interacting with both hydroxyl groups of EMB and with the terminal carboxylic acid groups of CIT, thus assisting in the solubilization of EMB. In Figures 7A,B similar density next to the amino groups of EMB could be assigned to CIT molecules (green arrow), with ARG being found in the close vicinity of EMB hydroxyl groups (yellow arrows). Water, although also interacting with the hydroxyl groups of EMB, is more frequently found in the first hydration shell surrounding ARG or CIT. Thus, data suggests that the presence of ARG confers additional stability to the internal liquid mixture structure by engaging intermolecular interactions with several EMB and CIT molecules, as it can be seen by the identification of several clusters comprising 2–4 EMB molecules during the MD trajectory (data not shown, exemplified in Figure 7B). However, as CIT and ARG are highly functionalized molecules, the remaining groups are kept immersed in the aqueous environment and assist in the effective solubilization of the liquid mixture components.

### Molecular docking

The results presented in the above section provided valuable insights on the internal structure of the therapeutic liquid mixtures and which specific interactions between EMB, CIT, and ARG contribute to the formation of the eutectic system. However, another critical aspect concerning the therapeutic value of this liquid mixture resides on understanding how such systems may promote the incorporation of EMB into the mycobacterial cell.

It is known that the lipidic content in the mycobacterial cell wall, comprising both outer and inner membranes, is highly enriched in diacyl phosphatidylmannosides as Ac_2_PIM_2_ or Ac_2_PIM_6_, providing an efficient permeability barrier due to its low fluidity and slowing the passive influx of drugs (Sartain et al., 2011; Bansal-Mutalik and Nikaido, 2014). This is a particular question in which concerns EMB, that is expected to be positively charged at physiological pH. To that matter the recent publication of a mycobacterial ABC transporter (Rv1819c, PDB ID: 6TQE), described to be involved in the uptake of hydrophilic compounds through the inner mycobacterial membrane (Rempel et al., 2020), may provide an entry pathway for EMB which is worth to be explored.

**FIGURE 8:**
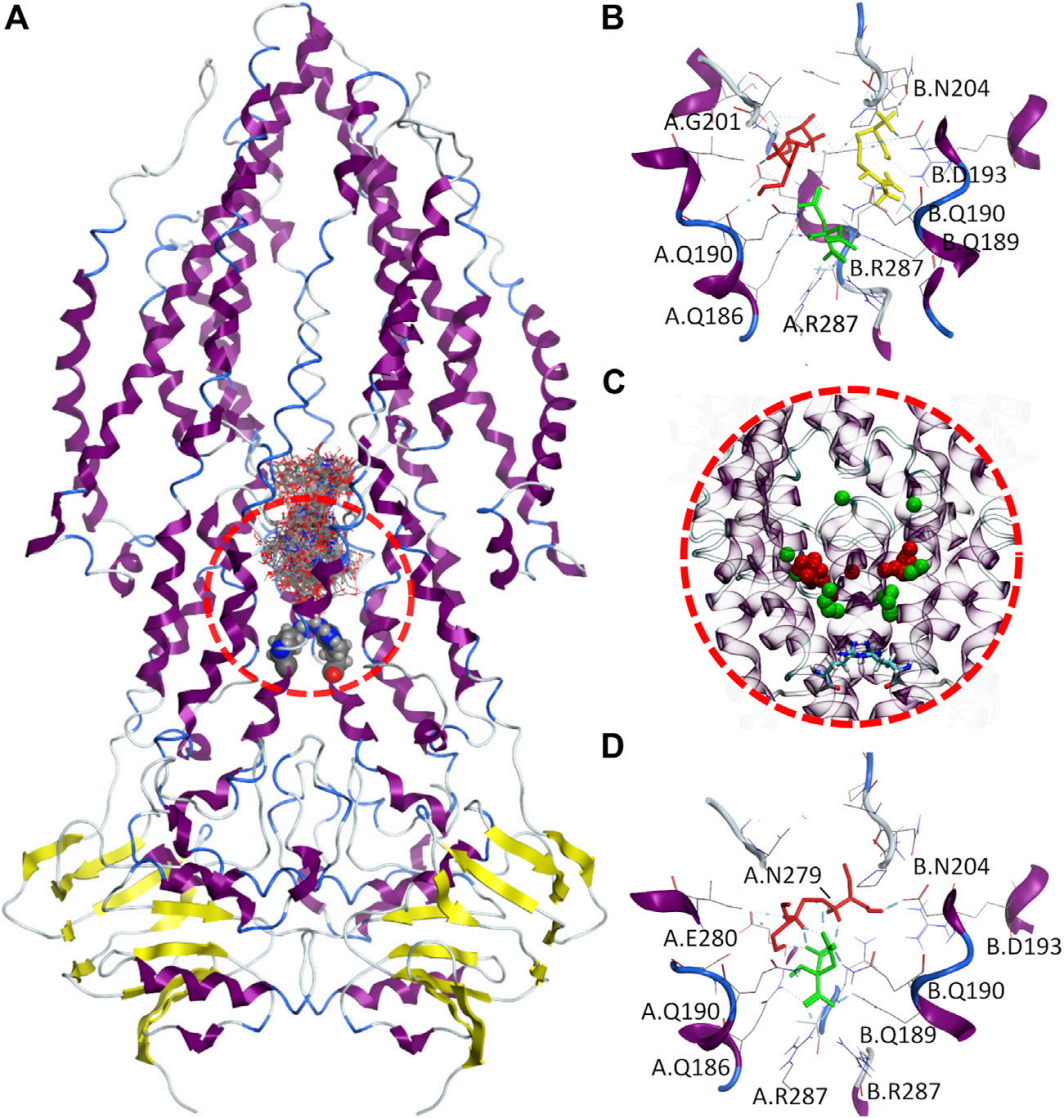
Molecular docking of therapeutic liquid mixtures components. **(A)** Final configuration for the 100 docking poses from multiple ligand simultaneous docking (MLSD) procedure, with gate residues (Arg287) depicted as volume spheres; **(B)** top-ranked docking poses for citric acid (green), arginine (yellow) and ethambutol (red) obtained with standard molecular docking; **(C)**, centers-of-mass for the top 20 poses from MLSD procedure for citric acid (green) and ethambutol (red), with gate residues (Arg287) depicted as licorice; and **(D)** top-ranked docking poses for citric acid (green) and ethambutol (red) from the MLSD procedure, with hydrogen-bonds depicted in cyan.

In this study, it was explored both single and multiple simultaneous ligands docking approaches to understand 1) if EMB can be internalized via Rv1819c, and 2) if any of the therapeutic liquid mixture components may enhance the interaction of EMB with the transporter. Due to the large internal cavity (∼7.700 Å³), we narrowed our search space to its lower section, defining a docking box ranging from a proposed intracellular gate formed by two R287 residues up to a 17-aminoacid loop in both transmembrane helix three that protrudes into the cavity (Figure 8A).

When EMB, ARG, and CIT are docked separately (Figure 8B), all clusters possess favorable binding energies (Table 2), but while CIT is found to be mostly located in the close vicinity of the R287, both EMB and ARG largest clusters are found in symmetrical locations, interacting with Q190/G201 in one protomer (EMB) or Q190/D193/N204 in the opposite protomer (ARG).

**TABLE 2:** Clustering results from single molecule molecular docking.

Therapeutic liquid mixture component	Number of docking poses in cluster	Lowest binding energy (Δ*G*_*bind*_)/kCal.mol⁻^1^	Ligand efficiency (LE)
**Ethambutol**	#1: 55	−3.61	−0.2579
	#2: 45	−3.28	−0.2343
**Citric acid**	#1: 98	−4.02	−0.3092
	#2: 2	−1.85	−0.1423
**Arginine**	#1: 57	−4.78	−0.3983
	#2: 41	−4.61	−0.3842
	#3: 1	−2.08	−0.1733
	#4: 1	−0.96	−0.0800

Interestingly, this changes when EMB and CIT are simultaneously docked. By visualizing the center-of-mass for both EMB and CIT obtained from the first 20 docking poses (Figure 8C), the results seem to suggest that most of the CIT molecules are located in the vicinity of the intracellular gate, interacting with R287 (cluster #1, 40 poses, Δ*G*_*bind*_−6.59 kCal.mol⁻^1^, LE −0.2441) or slightly above (cluster #2, 30 poses, Δ*G*_*bind*_−3.73 kCal.mol⁻^1^, LE −0.1381), but using its negatively charged carboxylate groups to strengthen the interactions between EMB and Rv1819c. A more detailed analysis of the interactions between the EMB/CIT top-ranked poses and the transporter corroborates this assumption. By establishing additional CIT/EMB intermolecular HBs (Figure 8D), the binding energies of the protein-ligand complex show a difference of only +1.64 kCal.mol⁻^1^ when compared with EMB and CIT binding energies obtained with the standard docking approach. Moreover, additional interactions with N279 and E280 may also contribute to decrease the binding energy of the protein-ligand complex. When considering the estimated dissociation constants (*K*_*d*_) this corresponds to a ∼150-fold decrease, from 2.26 mM (EMB) to only 14.85 µM (EMB/CIT complex), which means that internalization of EMB via Rv1819c would be favored in the presence of CIT.

We evaluated additional combinations through MSLD, for comparison purposes. For instance, replacing CIT by ARG slightly improves its binding affinities (cluster #1, 50 poses, ΔG_*bind*_ −6.79 kCal.mol⁻^1^, LE −0.2612; cluster #2, 34 poses, ΔG_*bind*_−6.04 kCal.mol⁻^1^, LE −0.2323), but again no molecules were found in the vicinity of R287 and no intermolecular interactions were found within the first 20 poses (except for pose 9). In another combination, including a second CIT, the binding affinity sharply decreases (cluster #1, 48 poses, ΔG_*bind*_−2.94 kCal.mol⁻^1^, LE −0.0735) and no significant intermolecular HBs were observed. Nonetheless, in the presence of an additional ARG molecule lower binding energies are restored and even improved (cluster #1, 21 poses, ΔG_*bind*_−8.26 kCal.mol⁻^1^, LE −0.1588; cluster #2, 14 poses, Δ*G*_*bind*_−7.48 kCal.mol⁻^1^, LE −0.1438; cluster #3, 32 poses, ΔG_*bind*_−5.51 kCal.mol⁻^1^, LE −0.1060). Again, intermolecular HBs between CIT/EMB/ARG, either in the vicinity of R287 (CIT/ARG) or next to the protruding loops (CIT/EMB) (clusters #1 and #2) or, alternatively, with a single CIT/EMB pair similar to that previously observed (cluster #3). Last but more important, *K*_*d*_ further increases to the nanomolar range (884.65 nM). A ∼15-fold increase was observed when compared with the one reported for the CIT/EMB complex.

Hence, our data suggests that the presence of CIT and/or ARG may also have an important role for EMB uptake by mycobacteria, apart from the prevention of EMB aggregation when present in the liquid mixture. Herein, the exploitation of a possible entry pathway through the ABC importer Rv1819c using single and multiple simultaneous ligand docking suggests that CIT may have a critical role in increasing the binding affinity of EMB towards Rv1819c by mediating specific interactions with its intracellular gate, formed by an ARG pair from both protomers (R287). Although we present a valid route for the uptake of EMB by mycobacteria, other entry routes, e.g. via direct interaction with the lipidic membrane or by means of other undisclosed transporters, cannot be discarded.

## Conclusion

Molecular dynamics was applied in this work to understand the internal structure and the molecular interactions that occur with the different components of the therapeutic liquid mixtures combining ARG, EMB, CIT and H_2_O. It was observed that EMB in water tends to aggregate, but when present in the liquid mixture with CIT and water it is stabilized. The radial distributions functions and spatial distribution functions calculated for different systems demonstrated that different ratios of CIT in the mixtures S2 and S3 could promote additional interactions by hydrogen-bonding in the system S3. In the system S4, with one more component (ARG), the data suggested that the structure of the liquid mixtures was more stable, since stronger intermolecular interactions could be formed due to the ability of ARG to disrupt the water-mediated hydrogen-bond network while promoting the hydrogen-bonding with the other components of the mixture. The MD data of these therapeutic liquid mixtures corroborates that while the carboxyl groups of CIT establish intermolecular interactions with amine groups of EMB, when ARG is present in the liquid mixtures it prefers to interact with hydroxyl groups of EMB and contributing to the solubility of EMB and ARG in water.

Furthermore, from molecular docking, different approaches were used to observe if the internalization of EMB via the mycobacterial ABC transporter (Rv1819c) is propitious by the liquid mixtures, and if the presence of CIT and ARG in the systems increases the affinity to the transporter. According to our findings, the different ratios of CIT in the mixtures did not had a direct impact in the EMB affinity to the binding site of the transporter, but nonetheless an important role for CIT as a mediator for EMB interaction with Rv1819c could also be hypothesized.

This computational approach applied to these therapeutic liquid mixtures represents an important step to understand molecular interactions that can be established between the different components of these low melting transition liquid mixtures, and why they could promote some modifications in properties of active compounds that are present in the mixtures. This will certainly be important to facilitate the entry of the anti-tuberculosis drug EMB in the mycobacterial cell.

## Data Availability

The original contributions presented in the study are included in the article/Supplementary Material, further inquiries can be directed to the corresponding authors.

## Abbreviations

MDR, multidrug resistance; LTTMs, low transition temperature mixtures; HBDs, hydrogen bond donors; HBAs, hydrogen bond acceptors; MD, molecular dynamics; API, active pharmaceutical ingredient; EMB, ethambutol; ARG, arginine.CIT citric acid; PBC, periodic boundary conditions; RDF, radial distribution functions; SDF, spatial distribution functions; MLSD, multiple ligand simultaneous docking; GA, genetic algorithm; HBs, hydrogen bonds.
